# Effects of marine-derived and plant-derived omega-3 polyunsaturated fatty acids on erythrocyte fatty acid composition in type 2 diabetic patients

**DOI:** 10.1186/s12944-022-01630-0

**Published:** 2022-02-10

**Authors:** Hechun Liu, Feng Wang, Xiaosong Liu, Yulan Xie, Hui Xia, Shaokang Wang, Guiju Sun

**Affiliations:** 1 Key Laboratory of Environmental Medicine and Engineering of Ministry of Education, and Department of Nutrition and Food Hygiene, School of Public Health, Southeast University, 87 Ding Jia Qiao Road, Nanjing, 210009 China; 2 Tianjin Institute of Environmental and Operational Medicine, 1 Da Li Road, Tianjin, 300050 China; 3 Guanlin Hospital, 17 Wenwei Road, Yixing, 214251 China; 4 Zhongda Hospital Southeast University, 87 Ding Jia Qiao Road, Nanjing, 210009 China

**Keywords:** Type 2 diabetes, Glucose, Polyunsaturated fatty acids, Erythrocyte

## Abstract

**Background:**

Dietary fatty acids intake affects the composition of erythrocyte fatty acids, which is strongly correlated with glycolipid metabolism disorders. This study aimed at investigating the different effects of marine-derived and plant-derived omega-3 polyunsaturated fatty acid (n-3 PUFA) on the fatty acids of erythrocytes and glycolipid metabolism in patients with type 2 diabetes mellitus (T2DM).

**Methods:**

The randomized double-blinded trial that was performed on 180 T2DM patients. The participants were randomly assigned to three groups for the six-month intervention. The participants were randomly assigned to three groups for the six-month intervention. The fish oil (FO) group was administered with FO at a dose of 3 g/day containing eicosapentaenoic acid (EPA) and docosahexaenoic acid (DHA), the perilla oil (PO) group was administered with PO at a dose of 3 g/day containing α-linolenic (ALA), the linseed and fish oil (LFO) group was administered with mixed linseed and fish oil at a dose of 3 g/day containing EPA, DHA and ALA. Demographic information were collected and anthropometric indices, glucose and lipid metabolism indexes, erythrocyte fatty acid composition were measured. Statistical analyses were performed using two-way ANOVA.

**Results:**

A total of 150 patients finished the trial, with 52 of them in the FO group, 50 in the PO group and 48 in the LFO group. There were significant effects of time × treatment interaction on fast blood glucose (FBG), insulin, HOMA-IR and C-peptide, TC and triglyceride (TG) levels (*P* < 0.001). Glucose and C-peptide in PO and LFO groups decreased significantly and serum TG in FO group significantly decreased (*P* < 0.001) after the intervention. Erythrocyte C22: 5 n-6, ALA, DPA, n-6/n-3 PUFA, AA/EPA levels in the PO group were significantly higher than FO and LFO groups, while EPA, total n-3 PUFA and Omega-3 index were significantly higher in the FO and LFO groups compared to PO group.

**Conclusion:**

Supplementation with perilla oil decreased FBG while fish oil supplementation decreased the TG level. Marine-based and plant-based n-3 PUFAs exhibit different effects on fatty acid compositions of erythrocytes and regulated glycolipid metabolism.

**Trial registration:**

This trial was recorded under Chinese Clinical Trial Registry Center (NO: ChiCTR-IOR-16008435) on May 28 2016.

## Introduction

Type 2 Diabetes mellitus (T2DM) is a major chronic diseases that is characterized by elevated glucose levels and insulin resistance in the body [1]. In 2019, it was postulated that the global number of adults (20–79 years) with diabetes was 463 million. This figure is projected to increase by 51% to reach 700 million by the year 2045 [2]. More than half of the T2DM population were in the high-risk status of dyslipidemia and vascular disease complications [3]. A reasonable diet structure is key for T2DM prevention and treatment. T2DM patients are frequently associated with dyslipidemia, which results in atherosclerosis (AS) and cardiovascular diseases (CVD). Approximately 80% of T2DM patients in the USA suffer from dyslipidemia [4].

Omega-3 polyunsaturated fatty acids (n-3 PUFA) are irreplaceable fatty acids for human from fish oils that contains eicosapentaenoic acids (EPA) and docosahexaenoic acid (DHA). In addition to this marine-based n-3 PUFA, α-Linolenic acid (ALA) is a plant-based n-3 PUFA that is an essential precursor of the n-3 PUFA family. Researches have explored the beneficial effects of major marine-based n-3 PUFA on human health, particularly on lipid profiles and glucose metabolism [5, 6]. Due to the poor palatability of fish oil and heavy metal pollution, the search for alternative plant-based n-3 PUFA sources is important [7].

Dietary n-3 PUFA is the most important determinant for balancing fatty acid status in the human body [8]. Fatty acid composition changes such as increased saturated fatty acids (SFA) and n-6/n-3 PUFA ratios are health risks for diabetes and dyslipidemia [9–12]. Fatty acids are involved in the de novo lipogenesis pathway, which is the key pathway in the pathogenesis of T2DM [13]. Previous studies have established that changes in serum, plasma, and erythrocyte fatty acids are biomarkers of diet exposure. However, erythrocytic changes reflect n-3 PUFA intake from food and metabolic conversion in the last 90 days. The conversion ratios from plasma to erythrocytes are stable [14].

Studies on fatty acid composition and their association with different diseases have focused on serum and plasma fatty acids. A few cross-sectional studies have been performed to investigate the biomarkers for n-3 PUFA in erythrocytes of diabetic and CVD patients. Only two RCT explored the effects of n-3 PUFA from different sources on erythrocyte n-3 fatty acid composition. These studies established that the blood glucose and lipid levels were within normal ranges. They focused on the n-3 PUFA of the erythrocyte but not SFA, monounsaturated fatty acids (MUFA), or n-6 PUFA [15, 16].

Our previous research found that fish oil supplement for 6 months improved serum EPA, DHA and TG levels inT2DM patients. The hypothesis of this trial was that plant n-3 PUFA supplement has the different effects on glucolipid metabolism in T2DM patients. Therefore, a double-blind, randomized trial with three groups was performed. The purpose was to compare the different effects of marine-based and plant-based n-3 PUFA on glucose, lipids profiles and erythrocyte fatty acids compositions in T2DM patients.

## Materials and methods

### Study participants

**Fig. 1:**
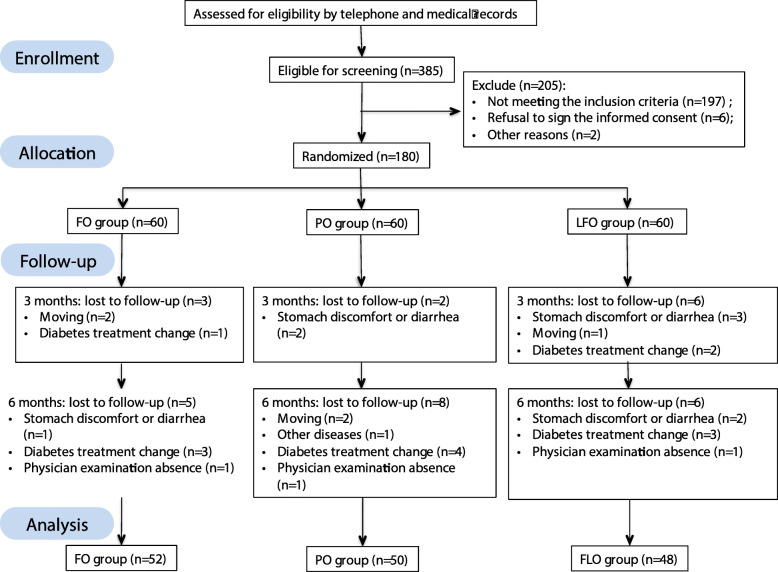
Flow chart of the randomized controlled trial

One hundred fifty type 2 diabetic and dyslipidemia people aged between 18 to 70 years were recruited from among 180 patients in Guanlin Public Hospital, Yixing City, China. The inclusion criteria were that the participants had to have been diagnosed with T2DM that fasting blood glucose (FBG) ≥ 7.0 mmol/L and dyslipidemia (TG ≥1.7 mmol/L, or TC ≥5.2 mmol/L, or LDL-C ≥ 3.4 mmol/L, or HDL-C < 1.0 mmol/L, or no HDL-C ≥ 4.1 mmol/L). Patients who had been on omega-3 supplements within 6 months or on lipid-lowering drugs, those with complicated chronic cardiovascular diseases, asthma, alcoholism, hyperthyroidism, tumor patients, pregnancy, lactation period were excluded from the study. Finally, the physical examination of patients for participation in the study was done by a competent physician. Figure 1 shows the process of participant selection and allocation in the trial.

Using the formula *N* = 2(*Zα* + *Zβ*)^2^*σ*^2^/*d*^2^ for sample size calculation, 48 individuals were required per group in this trial. According to the previous trial and reported literature, 0.5 mmon/L, 0.3 mmon/L and 0.4 mmon/L was set up as the TG decreasing mean after fish oil, perilla oil and blend oil intervention, respectively. The standard deviations were 0.3 mmon/L, 0.2 mmon/L, 0.3 mmon/L respectively [17, 18]. With α = 0.05 and β = 0.1, at least 48 subjects were needed per group. According to the 20% lost to follow-up, 180 participants were selected for final inclusion.

The trial was approved by the Ethical Committee of Zhongda hospital affiliated to Southeast University, Nanjing, China (NO. 2015ZDSYLL089.0). The purpose and protocol of the trial were exhaustively explained to the study participants. All participants were required to sign the informed consent before enrollment. The trial was recorded under http://www.chictr.org.cn/showprojen.aspx?proj=14291 (ChiCTR-IOR-16008435).

### Experimental oil capsule preparation

The fish oil (FO), perilla oil (PO), or mixed linseed and fish oil (LFO) capsules were standardized to 500 mg. Each FO capsule contained 143 mg EPA and 172 mg DHA. Each PO capsule contained 322 mg of ALA. The major fatty acids in each LFO capsule were EPA (105 mg), DHA (60 mg) and ALA (140 mg). These capsules were manufactured by the Zhanwang Company (Shanghai, China). They exhibited the same shape and were packaged in transparent bottles. The bottles were labeled with ‘PUFA A’, ‘PUFA B’ and ‘PUFA C’ for FO, PO and LFO, respectively. Fatty acid contents of intervention capsules were shown in Table 1. Except for the analyzer, participants, nurses and outcomes assessor were blinded during the intervention.

**Table 1:** Fatty acid contents of intervention capsules (%)

Fatty acids (%)	Perilla oil capsules	Fish oil capsules	Linseed and fish oil capsules
<C14	——(< 0.0013)	——(< 0.0013)	——(< 0.0013)
C14:0	——(< 0.0013)	——(< 0.0013)	——(< 0.0013)
C16:0	5.6	5.6	5.6
C17:0	0.0425	0.0425	0.0425
C17:1	——(< 0.0013)	——(< 0.0013)	——(< 0.0013)
C18:0	2.29	2.29	2.29
C18:1	13.7	13.7	13.7
C18:2	14	14	14
C18:3 n3	63.4	——(< 0.0066)	28
C20:0	0.108	0.108	0.108
C20:1	——(< 0.0013)	——(< 0.0013)	——(< 0.0013)
C20:2	——(< 0.0013)	——(< 0.0013)	——(< 0.0013)
C20:5 n3	——(< 0.0066)	28.6	21
C22:0	——(< 0.0026)	——(< 0.0026)	——(< 0.0026)
C22:1	——(< 0.0013)	——(< 0.0013)	——(< 0.0013)
C22:6 n3	——(< 0.0066)	34.4	12
C24:0	——(< 0.0026)	——(< 0.0026)	——(< 0.0026)

—— not detected

### Omega-3 fatty polyunsaturated fatty acid intervention

After the baseline examination and stratified by gender and age, patients were randomly distributed in three groups (at a 1:1:1 ratio) by reference to the computer-generated random number list, with 60 patients in each group. The intervention duration was 6 months. FO group participants were administered with two capsules of fish oil three times a day; PO group participants were administered with perilla oil at the same dose; LFO group participants were administered with mixed linseed oil and fish oil capsules. During the experimental period, subjects were instructed to maintain their habitual dietary behaviors and lifestyles.

### Questionnaire survey and physical evaluation

The unified questionnaires were used to obtain information on demographic characteristics, diseases history, diabetes duration, treatment modality, smoking status and alcohol intake. Physical evaluation was performed. Blood pressure was measured twice for each subject (Yuwell medical equipment & supply Co., Ltd., Jiangsu, China).

### Biochemical analysis

Overnight fasting venous blood samples were obtained between 7:00 am to 9:00 am into vacationer tubes containing ethylenediaminetetraacetic acid (EDTA). C peptide and insulin were measured by an electrochemiluminescence analyzer (Roche Cobas e 602). FBG levels and TG, TC, HDL-C, LDL-C lipoprotein a (LPa), apolipoprotein A1 (Apo A1), apolipoprotein B (Apo B), free fatty acids (FFA), interleukin 6 (IL-6), markers of hepatic or renal function were determined by an automatic blood biochemical analyzer (Mindray BS-800, Shenzhen, China). Glycated haemoglobin (HbAlc) was determined by chromatography (Audicom, AC6601, Jiangsu, China).

### Lipid and erythrocyte fatty acid analysis

Approximately 7 mL fasting blood was obtained from forearm veins. After centrifugation at 3000 rpm/min for 15 min, red blood cells were separated from the serum. Lipids were extracted from the erythrocyte by chloroform/methanol (2:1) solution with 10 mg/L butylated-hydroxytoluene. The total weight of lipids was determined by gravimetric analysis. Fatty acid content and composition in the erythrocyte were analyzed by gas chromatography (GC) after total lipid extraction. Toluene and H_2_SO_4_ in methanol (5%, v/v) were used for the transesterification of erythrocyte fatty acid methyl esters in sealed tubes at 70 °C for 2 h. FAME was analyzed using a Shimadzu GC-14C gas chromatography-flame ionization detector (Shimadzu Corporation, Japan). The GC column was a DB-23 fused silica bonded phase column 60 m with 0.25 mm diameter and 0.25 μm film thickness (Agilent Corporation, California, USA). The column temperature was programmed as follows: the initial temperature was 150 °C increased to 180 °C at a rate of 10 °C/min and held for 2 min; then further increased to 215 °C and maintained for 6 min. It was finally raised to 230 °C followed by a 5 min hold time. The retention time and FAME mixture standards (Nu-Chek Prep, MN, USA) were used to identify individual fatty acids. Before extraction, 1 mg internal standard was added to each 500 mg samples. The relative quantities of individual fatty acids were expressed as a percentage of total fatty acids in the erythrocytes based on the peak area.

### Statistical analysis

Survey data and testing results were double-entered by two independent entry clerks into the EpiData 3.1. Data were checked for consistency. Statistical calculations were conducted by the SPSS software system (version 22.0). The present report was based on the pre-protocol analysis. If the data met the normal distribution and homogeneous variance, they were expressed as mean ± SD and two-way ANOVA was performed to test statistical significance among three groups, followed by Tukey’s post hoc test. If the data were not homogenous, it was expressed as median (25, 75% quartile) and Kruskal-Wallis test was performed to test statistical significance among three groups comparisons, followed by Dunn’s test. The two-factor repeated ANOVA was performed to determined the treatment-by-time interaction effects on all outcomes, with baseline as covariates. Categorical variables were expressed as counts. The paired sample t-test was performed for the comparison of before and after intervention in the same group. Chi-square test was performed for counting data. *P*-value ≤0.05 was set as the threshold for statistical significance. Non HDL = TC − HDL. $\mathrm{HOMA}-\mathrm{IR}=\left( Fasting\ Glucose\ \left(\frac{mmol}{L}\right)\times insulin\left(\frac{mU}{L}\right)\right)/22.5$.

## Results

### Baseline characteristics

As shown in Fig. 1, a total of 150 participants completed the trial, with 52 of them in the fish oil group, 50 in the PO group and 48 in the LFO group. Table 2 shows the anthropometric characteristics of the three groups of participants. The mean age was 62.45 ± 8.58 years old and the mean BMI was 26.58 ± 3.23 kg/m^2^ with 67.3% of the study participants’ exhibiting excess body weight. At the beginning of the study, age, gender composition, diabetes duration, treatment programme, smoking habits, alcohol consumption, body weight, BMI, waist circumference, hip circumference did not exhibit significant differences among the three groups. The mean BMI was 26.58 ± 3.23 kg/m^2^ with 67.3% of the study participants’ exhibiting excess body weight. The levels of markers of hepatic or renal function were not different among the FO, PO and LFO groups. The renal and liver functions were all normal. After 6 months of intervention, both SBP and DBP were significantly decreased (Table 3).

**Table 2:** Main Characteristic of subjects completing trial at baseline

Variables	Total sample	FO (*n* = 52)	PO (*n* = 50)	LFO (*n* = 48)	*p*-value
Age (years)	62.45 ± 8.58	62.29 ± 7.55	63.84 ± 9.67	61.19 ± 8.38	0.308 ^a^
Sex (male/female, n)	57/93	17/35	20/30	20/28	0.612 ^b^
Diabetes duration (years)	7 (3, 11)	8 (4, 11)	7 (3, 13)	8 (3, 10)	0.890 ^c^
Diabetes treatment					0.599 ^b^
• Oral hypoglycemic agents (n)	83	27	32	24	
• Insulin injection (n)	22	8	4	10	
• Two items above (n)	38	15	11	12	
• Diet controlled (n)	7	2	3	2	
Smoking habits (male/female, n)					0.295 ^b^
• Never	25/91	8/34	7/30	10/27	
• Previous	3/2	2/1	0/0	1/1	
• Current	29/0	7/0	13/0	9/0	
Alcohol consumption (n)					
• Never	113	37	39	37	0.280 ^b^
• Alcohol less than once per week	4	1	3	0	
• Alcohol once a week or more	33	14	8	11	
Weight (cm)	67.90 ± 10.26	66.34 ± 9.59	69.27 ± 10.58	68.13 ± 10.61	0.353 ^a^
Height (cm)	159.72 ± 8.61	158.40 ± 8.40	160.14 ± 8.33	160.69 ± 9.11	0.384 ^a^
Body mass index (kg/m^2^)	26.58 ± 3.23	26.38 ± 2.68	26.99 ± 3.44	26.38 ± 3.56	0.560 ^a^
Overweight n(%)	101 (67.3%)	17 (32.7%)	14 (28%)	18 (37.5%)	0.605 ^b^
Waist circumference (n)	91.63 ± 8.05	90.18 ± 7.27	91.48 ± 8.50	93.26 ± 8.21	0.155 ^a^
Hip circumference (n)	100.42 ± 5.90	100.18 ± 5.34	101.54 ± 6.57	99.50 ± 5.67	0.218 ^a^
Waist-to-hip ratio	0.91 ± 0.05	0.90 ± 0.05	0.92 ± 0.06	0.92 ± 0.06	0.138 ^a^
ALT (U/L)	24.36 ± 11.46	24.66 ± 13.26	24.23 ± 10.73	24.17 ± 10.27	0.973 ^a^
AST (U/L)	22.22 ± 8.96	22.40 ± 9.87	22.13 ± 5.71	22.11 ± 10.72	0.984 ^a^
Creatinine (umol/L)	61.83 ± 13.08	62.51 ± 14.67	60.20 ± 12.19	62.81 ± 12.23	0.559 ^a^
Uric acid (umol/L)	329.64 ± 83.85	313.94 ± 85.88	342.27 ± 88.09	333.50 ± 75.75	0.218 ^a^
Urea nitrogen (mmol/L)	6.19 ± 1.54	6.13 ± 1.33	6.40 ± 1.73	6.05 ± 1.54	0.482 ^a^
Total protein (g/L)	77.35 ± 3.52	77.50 ± 3.35	77.56 ± 3.21	76.97 ± 4.02	0.663 ^a^
Albumin (g/L)	46.91 ± 2.27	46.80 ± 2.18	47.09 ± 2.37	46.83 ± 2.30	0.784 ^a^
Globulin (g/L)	30.45 ± 3.46	30.70 ± 3.48	30.47 ± 3.18	30.14 ± 3.75	0.782 ^a^

a Data are mean ± standard deviation, ANOVA test for comparing difference among groups;

b Data are presented as number (%), χ2 test for comparing difference among groups;

c Data are presented as median (P25, P75), Kruskal-Wallis tests for comparing difference among groups

*FO* Fish oil, *PO* Perilla oil, *LFO* Mixed linseed oil and fish oil; ALT glutamic-pyruvic transaminase; AST glutamic-oxalacetic transaminease

**Table 3:** Blood pressures at baseline and after 6-months of each treatment

Blood Pressures	FO		PO		LFO		T Effect	t Effect	T × t Interaction
	Baseline	6 month	Baseline	6 month	Baseline	6 month	*p*	*p*	*p*
SBP (mm Hg)	144.78 ± 18.86	139.23 ± 18.68	148.36 ± 18.19	139.04 ± 16.34	143.02 ± 18.53	139.13 ± 17.10	0.998	< 0.001	< 0.001
DBP (mm Hg)	85.76 ± 9.11	80.77 ± 8.18	87.36 ± 10.41	82.36 ± 8.22	88.48 ± 8.33	83.42 ± 7.73	0.256	< 0.001	< 0.001

T: treatment effect. ANOVA test for comparing difference among groups;

t, time effect. Paired t test for comparing differences before and after intervention;

T × t: treatment×time interaction effect. Two-way repeated ANOVA test for assess the interaction effects adjusted the baseline values of each biochemical variables as covariate

Values are expressed as mean ± SD

*FO* Fish oil, *PO* Perilla oil, *LFO* Mixed linseed oil and fish oil, *SBP* Systolic blood pressure, *DBP* Diastolic blood pressure

### Blood biochemical indices

**Fig. 2:**
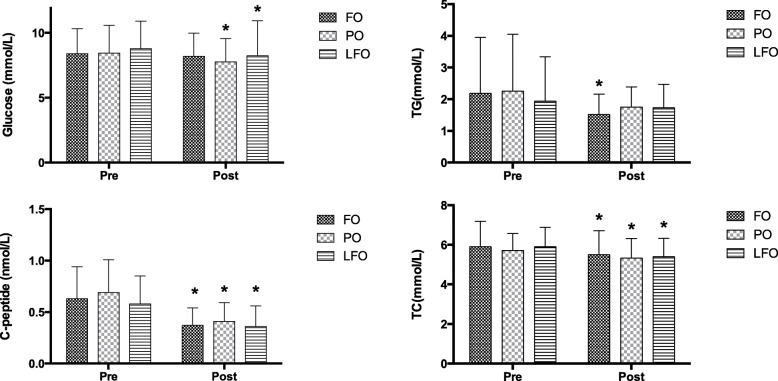
Main glucose and lipid index at baseline and after 6-month of each treatment. * Significantly different as compared to baseline (*p* < 0.05). Values are expressed as mean ± SD. FO fish oil; PO perilla oil; LFO mixed linseed oil and fish oil; TG Triglyceride; TC total cholesterol

The serum glucose and lipid profiles are presented in Table 4 and Fig. 2. There were no statistical differences in glucose indices and lipid profiles among groups before treatments. Two-way ANOVA showed statistical effects of time × treatment interaction on FBG, insulin, HOMA-IR and C-peptide. Administration of PO and LFO significantly decreased the FBG and glycated hemoglobin (HbA1c) levels. Administration of all n-3 PUFA significantly reduced insulin and C-peptide concentrations in the three groups after treatments. The significant time × treatment interaction effects for lipid profiles, FFA and IL-6 were observed (*P* < 0.001). Administration of FO significantly decreased serum TG levels and TG/HDL ratio. Serum TC, Apo A1 and IL-6 levels in the all of the treatment groups decreased after the interventions.

**Table 4:** Glucose and lipid index at baseline and after 6-month of each treatment

Blood biochemical index	FO (*n* = 52)		PO (*n* = 50)		LFO (*n* = 48)		T Effect	t Effect	T × t Interaction
	Baseline	6 month	Baseline	6 month	Baseline	6 month	*p*	*p*	*p*
Glucose (mmol/L)	8.38 ± 1.94	8.19 ± 1.80	8.43 ± 2.15	7.76 ± 1.79 *	8.77 ± 2.13	8.24 ± 2.69 *	0.464	0.005	< 0.001
Insulin (mU/L)	7.84 (4.64,13.66)	3.97 (2.44,6.93) *	9.42 (5.63,14.06)	4.77 (2.96,8.82) *	9.45 (5.60,15.21)	4.28 (2.52,14.84) *	0.656	< 0.001	< 0.001
HOMA-IR ^a^	2.93 (1.76,5.36)	1.45 (0.78,2.53) *	3.39 (1.94, 4.94)	1.60 (0.87,3.42) *	3.35 (2.22, 5.66)	1.41 (0.87,4.87)	0.454	< 0.001	< 0.001
C-peptide (nmol/L)	0.63 ± 0.31	0.37 ± 0.17 *	0.69 ± 0.32	0.41 ± 0.18 *	0.58 ± 0.27	0.36 ± 0.20 *	0.311	< 0.001	< 0.001
HbA1c	6.79 ± 1.05	6.54 ± 1.19	6.94 ± 1.09	6.62 ± 1.29 *	6.74 ± 1.09	6.27 ± 1.17 *	0.324	< 0.001	< 0.001
TG (mmol/L)	2.18 ± 1.77	1.52 ± 0.64 *	2.25 ± 1.80	1.75 ± 0.64	1.94 ± 1.40	1.73 ± 0.74	0.227	0.324	< 0.001
TC (mmol/L)	5.91 ± 1.28	5.50 ± 1.21 *	5.72 ± 0.85	5.33 ± 0.99 *	5.91 ± 0.98	5.40 ± 0.92 *	0.697	< 0.001	< 0.001
HDL-C (mmol/L)	1.28 ± 0.32	1.36 ± 0.29	1.25 ± 0.24	1.24 ± 0.23	1.30 ± 0.30	1.28 ± 0.30	0.083	0.980	< 0.001
LDL-C (mmol/L)	2.83 ± 0.99	2.90 ± 0.76	2.74 ± 0.60	2.57 ± 0.57 *	2.99 ± 0.71	2.91 ± 0.73	0.022	0.661	< 0.001
non HDL-C (mmol/L)	4.63 ± 1.20	4.18 ± 1.18 *	4.46 ± 0.80	4.11 ± 1.00 *	4.61 ± 0.84	4.12 ± 0.79 *	0.926	< 0.001	< 0.001
LPa (mg/L) ^a^	121.23 (54.63, 406.75)	138.95 (61.66,334.77)	197.58 (62.57,410.12)	132.45 (44.77,308.22) *	175.61 (62.94,326.93)	134.90 (61.52,277.00) *	0.997	< 0.001	< 0.001
Apo A1 (g/L)	1.42 ± 0.24	1.34 ± 0.20 *	1.40 ± 0.18	1.33 ± 0.17 *	1.44 ± 0.21	1.34 ± 0.19 *	0.874	< 0.001	< 0.001
Apo B (g/L)	1.05 ± 0.27	1.07 ± 0.30	1.05 ± 0.20	0.95 ± 0.24 *	1.11 ± 0.21	1.06 ± 0.20 *	0.029	0.037	< 0.001
LDL/HDL ratio	2.24 ± 0.80	2.23 ± 0.60	2.23 ± 0.56	2.17 ± 0.52	2.34 ± 0.57	2.33 ± 0.59	0.385	0.982	< 0.001
TG/HDL ratio ^a^	1.33 (0.88,2.27)	1.04 (0.71,1.42) *	1.41 (0.89,2.27)	1.26 (0.95,1.90)	1.34 (0.72,2.20)	1.26 (0.77,1.99)	0.733	0.686	< 0.001
Apo A1/Apo B ^a^	1.40 (1.22,1.56)	1.28 (1.12,1.54)	1.35 (1.14,1.54)	1.36 (1.18,1.60)	1.27 (1.12,1.44)	1.31 (1.10,1.44)	0.313	0.488	0.475
FFA (mmol/L)	0.50 ± 0.24	0.45 ± 0.19	0.52 ± 0.23	0.54 ± 0.23	0.50 ± 0.25	0.43 ± 0.16	0.024	0.167	< 0.001
IL6 (mg/L)	10.20 ± 2.90	2.95 ± 1.44 *	10.05 ± 2.95	3.68 ± 2.47 *	9.76 ± 4.45	3.18 ± 2.15 *	0.188	< 0.001	0.003

a Data presented as median (P25, P75), Kruskal-Wallis tests for comparing difference among three groups

T: treatment effect. ANOVA test for comparing difference among groups;

t, time effect. Paired t test for comparing differences before and after intervention;

T × t: treatment×time interaction effect. Two-way repeated ANOVA test for assess the interaction effects adjusted the baseline values of each biochemical variables as covariate

* Significantly different as compared to baseline (p < 0.05). Values are expressed as mean ± SD

*FO* Fish oil, *PO* Perilla oil, *LFO* mixed linseed oil and fish oil, *HbA1c* Glycated hemoglobin, *HOMA-IR* Homeostasis model assessment of insulin resistance, *TG* Triglyceride, *TC* Total cholesterol, *HDL-C* High-density lipoprotein cholesterol, *LDL-C* Low-density lipoprotein cholesterol, *LPa* Lipoprotein a, *Apo A1* Apolipoprotein A1, *Apo B* Apolipoprotein B, *FFA* Free fatty acids, *IL-6* Interleukin

### Fatty acids composition of erythrocyte

**Fig. 3:**
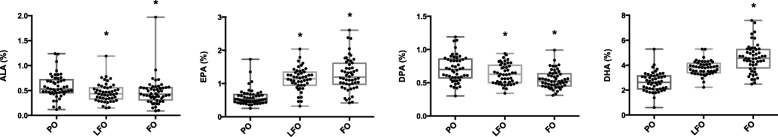
Main n-3 PUFA of erythrocyte after 6-months of each treatment. * significantly different as compared to PO (p < 0.05). FO fish oil; PO perilla oil; LFO mixed linseed oil and fish oil; ALA α-linolenic acid; EPA eicosapentaenoic acid; DPA docosapentaenoic acid; DHA docosahexaenoic acid

The fatty acids composition of erythrocyte was shown in Table 5 and Fig. 3. There were significant differences in C20:0, C22:5 n-6, ALA, EPA, C22:5 n-3 (docosapentaenoic acid, DPA), DHA, total n-6 PUFAs, total n-3 PUFAs, n-6 PUFAs/n-3 PUFAs ratio, arachidonic acid (AA)/EPA ratio and Omega-3 index among the three groups after adjusting the age, sex and free fatty acids levels. C22: 5 n-6, ALA, DPA, n-6/n-3 PUFAs, AA/EPA in the PO group were significantly higher, while EPA, total n-3 PUFAs and Omega-3 index were significantly lower in the PO group compared with FO and LFO groups. In addition, DHA was significantly higher in the FO than that in the PO group. In contrast, total n-6 PUFAs in FO was significantly lower than that in the PO. After intervention, similar trends were observed for total n-3 PUFAs and Omega-3 index in the three treatment groups; the FO was highest, followed by LFO and PO, while the n-6 PUFAs/n-3 PUFAs ratio had the opposite trend.

**Table 5:** Fatty acid compositions of erythrocyte after 6-months of each treatment

Fatty Acid (%)	PO	LFO	FO	*p*-value	Adjusted *p*-value ^a^
SFAs					
C14:0 (myristic acid) ^b^	0.21 (0.16, 0.28)	0.20 (0.15, 0.25)	0.19 (0.13, 0.27)	0.341	0.253
C15:0 (pentadecanoic acid)	0.29 ± 0.06	0.31 ± 0.06	0.30 ± 0.08	0.347	0.349
C16:0 (palmitic acid)	13.86 ± 1.57	14.22 ± 0.97	13.93 ± 1.17	0.322	0.149
C17:0 (margaric acid)	0.81 ± 0.18	0.87 ± 0.17	0.91 ± 0.17 *	0.019	0.091
C18:0 (stearic acid) ^b^	16.74 (15.72, 17.91)	17.09 (16.43, 17.71)	16.76 (15.62, 17.86)	0.387	0.575
C20:0 (arachidic acid)	0.24 ± 0.05	0.26 ± 0.07	0.27 ± 0.08 *	0.030	0.034
C21:0 ^b^	0.00 (0.00, 0.00)	0.00 (0.00, 0.00)	0.00 (0.00, 0.00)	0.250	0.293
C22:0 (behenic acid)	0.50 ± 0.18	0.55 ± 0.17	0.50 ± 0.16	0.238	0.303
C23:0 (tricosanoic acid) ^b^	0.00 (0.00, 0.00)	0.00 (0.00, 0.00)	0.00 (0.00, 0.00)	0.075	0.413
C24:0 (lignoceric acid)	1.22 ± 0.49	1.36 ± 0.49	1.20 ± 0.46	0.164	0.222
MUFAs					
C14:1	0.27 ± 0.38	0.20 ± 0.12	0.23 ± 0.12	0.321	0.147
C15:1 ^b^	3.23 (2.68, 3.65)	3.30 (2.87, 3.62)	3.09 (2.53, 3.52)	0.371	0.109
C16:1 ^b^	0.60 (0.41, 0.84)	0.50 (0.35, 0.79)	0.61 (0.41, 0.92)	0.321	0.506
C17:1	4.12 ± 1.37	4.25 ± 1.41	4.03 ± 1.38	0.721	0.578
C18:1 trans-9	0.27 ± 0.07	0.26 ± 0.04	0.25 ± 0.07	0.231	0.289
C18:1 cis-9 (oleic acid) ^b^	12.46 (11.38, 13.55)	12.04 (11.35, 17.70)	11.96 (11.33, 12. 90)	0.308	0.505
C20:1 n-9 (eicosenoic acid)	0.55 ± 0.26	0.55 ± 0.18	0.58 ± 0.33	0.750	0.713
C22:1 ^b^	0.15 (0.00, 0.35)	0.23 (0.00, 0.46)	0.18 (0.00, 0.44)	0.314	0.190
C24:1 n-9 (nervonic acid)	2.52 ± 1.16	3.20 ± 1.36 *	2.97 ± 1.54	0.043	0.080
n-6 PUFAs					
C18:2 trans-6	1.69 ± 0.64	1.59 ± 0.74	1.69 ± 0.66	0.696	0.609
C18:2 cis-6 (linoleic acid)	18.58 ± 2.66	17.81 ± 2.23	17.49 ± 2.69	0.084	0.167
C18:3 n-6 (γ-linolenic acid)	0.14 ± 0.07	0.14 ± 0.07	0.16 ± 0.07	0.544	0.408
C20:3 n-6	1.82 ± 0.76	1.65 ± 0.26	1.72 ± 0.34	0.203	0.337
C20:4 n-6 (Arachidonic acid)	12.03 ± 2.31	12.46 ± 1.71	11.73 ± 1.59	0.147	0.173
C22:2 n-6 ^b^	0.00 (0.00, 0.00)	0.00 (0.00, 0.00)	0.00 (0.00, 0.00)	–	0.540
C22:5 n-6 ^b^	0.71 (0.58, 0.85)	0.63 (0.51, 0.78) *	0.53 (0.46, 0.64) *	< 0.001	< 0.001
n-3 PUFAs					
C18:3 n-3 (α-linolenic acid)	0.58 ± 0.23	0.46 ± 0.18 *	0.46 ± 0.18 *	0.012	0.037
C20:3 n-3 ^b^	0.00 (0.00, 0.00)	0.00 (0.00, 0.00)	0.00 (0.00, 0.00)	0.618	0.681
C20:5 n-3 (eicosapentaenoic acid) ^b^	0.52 (0.45, 0.67)	1.13 (0.93, 1.34) *	1.23 (0.99, 1.62) *	< 0.001	< 0.001
C22:5 n-3 (docosapentaenoic acid) ^b^	0.71 (0.58, 0.85)	0.63 (0.51, 0.78) *	0.53 (0.46, 0.64) *	< 0.001	< 0.001
C22:6 n-3 (docosahexaenoic acid)	2.66 (2.10, 3.14)	3.80 (3.37, 4.10)	4.58 (3.93, 5.27) *	< 0.001	< 0.001
Others C20:2 (eicosadienoic acid)	0.60 ± 0.10	0.59 ± 0.07	0.60 ± 0.09	0.724	0.034
Fatty acids composition of erythrocytes					
Total SFAs	33.86 ± 3.00	34.90 ± 2.09	34.23 ± 2.75	0.138	0.129
Total MUFAs	26.27 ± 5.54	24.49 ± 2.30	25.10 ± 4.44	0.122	0.185
Total PUFAs	37.92 ± 3.36	38.76 ± 2.31	38.74 ± 3.49	0.298	0.460
Total n-6 PUFAs ^b^	33.35 (31.67, 34.69)	32.20 (30.74, 33.94)	30.95 (29.23, 34.04) *	0.019	0.050
Total n-3 PUFAs ^b^	4.56 (3.97, 5.03)	5.99 (5.57, 6.50) *#	6.71 (6.12, 7.82) *	< 0.001	< 0.001
n-6/n-3 PUFA ^b^	7.17 (6.69, 8.42)	5.31 (4.88, 5.88) *#	4.59 (3.88, 5.20) *	< 0.001	< 0.001
AA/EPA	22.68 ± 7.93	13.18 ± 7.86 *	10.94 ± 6.13 *	< 0.001	< 0.001
MUFAs/SFAs ratio ^b^	0.73 (0.68, 0.78)	0.69 (0.65, 0.76)	0.72 (0.63, 0.81)	0.253	0.124
PUFAs/SFAs ratio	1.12 ± 0.10	1.12 ± 0.11	1.14 ± 0.13	0.636	0.542
Omega-3 index(%) ^b^	3.24 (2.66, 3.70)	4.86 (4.50, 5.38) *#	5.70 (5.06, 6.91) *	< 0.001	< 0.001

Data are mean ± standard deviation, ANOVA test for comparing difference among groups, followed by Tukey’s pos hoc test

a ANOVA test for comparing difference among groups after adjusting the age, sex and free fatty acids levels

b Data presented as median (P25, P75), Kruskal-Wallis tests for comparing difference among groups, followed by Dunn’s test

* significantly different as compared to PO (*p* < 0.05)

# significantly different as compared to FO (*p* < 0.05)

*FO* Fish oil, *PO* Perilla oil, *LFO* Mixed linseed oil and fish oil, *SFA* Saturated fatty acid, *MUFA* Molyunsaturated fatty acid, *PUFA* Polyunsaturated fatty acid, *AA* Arachidonic acid, *EPA* Eicosapentaenoic acid

## Discussion

For diabetic patients, it remains unknown whether different sources of n-3 PUFA have the same effects on glucose and lipid metabolism. The present trial compared the different effects of marine-based n-3 PUFA and plant-based n-3 PUFA on the glucose and lipid profiles. It was found that administration of marine-based n-3 PUFA significantly increased erythrocyte EPA, DHA, total n-3 PUFA levels and omega-3 index compared to plant-derived n-3 PUFA intake. However, administration of plant-derived n-3 PUFA significantly increased C22:5n-6, ALA and DPA levels of erythrocyte significantly. Moreover, both marine and plant-derived n-3 PUFA administration reduced insulin, C-peptide and serum TC levels in T2DM patients. There was a decrease in serum TG after administration of marine-based n-3 PUFAs. There was the significant decrease in FBG after the administration of plant-derived n-3 PUFA.

Most studies have indicated that n-3 PUFA especially marine-based n-3 PUFA intake results in favorable lipid profiles. Many meta-analyses showed that EPA and DHA consumption can significantly reduce the levels of TG, LDL in T2DM patients [5, 19]. And n-3 PUFA intake for both ≤8 and > 8 weeks could significantly increase the HDL level [20]. It is well established that T2DM is associated with dyslipidemia and that EPA and DHA supplementation has long been indicated in the treatment of hyperlipidemia in T2DM and pre-diabetes patients. The beneficial actions of n-3 PUFA on inflammation have been reported in patients with high inflammatory states [21, 22]. Fish oil and ALA have also been shown to exhibit anti-inflammatory effects [23–25]. Although the meta-analysis indicated that the reduction in the c-reactive protein were not statistically significant [20, 26]. The reduction of IL-6 after EPA and DHA supplementation was significant in T2DM patients [27]. One recent cross-sectional study showed that DHA levels in erythrocytes was negatively related to the IL-6 and EPA levels in erythrocytes was negatively associated with TNF-α [28]. The aetiology of T2DM was associated with metabolic abnormality and inflammation. Therefore, the benefits of marine and plant n-3 PUFA may be not consistent in T2DM patients. EPA and DHA have the positive effects on lipid profile markers and may be more likely to improve dyslipidemia, which is one of the main risk factors and complications of T2DM. On the other hand, n-3 PUFA inhibited the pro-inflammatory cytokines and promoted resolution of inflammation, which will improve insulin resistance.

Supplementation with n-3 PUFA has protective effects on diabetes factors, while the effects of different sources n-3 PUFA on glycose were controversial. The present results showed that the influences of EPA and DHA vs ALA on FBG are different. Administration with EPA and DHA did not affect fasting glucose. These results are in tandem with two recent meta-analyses which showed no significant relationship between EPA and DHA supplementation and blood glucose level [5, 27]. Even EPA and DHA are associated statistically significantly with increased levels of FBG among T2DM patients in Natto et al’s meta-analysis. This analysis excluded studies that were administered less than 1000 mg/day of n-3 PUFA [26]. However, Delpino et al’s meta-analysis presented the opposite results, which was probably due to the inclusion of gestational diabetes studies [29]. Besides FBG, O′Mahoney’s study revealed that a small reduction in HbA1c following EPA and DHA supplementation. But sensitivity analysis showed that removal of two trials changed the statistical results from significant to non-significant [27]. And two meta-analyses also showed that the level change of HbA1c was not statistically significant [26, 29]. But in Khalili’s study, n-3 PUFA consumption including DHA, EPA and ALA can significantly reduce HbA1c (− 0.74 (− 1.13 to − 0.35)). The possible reason for these inconsistent results is that the n-3 PUFA sources were different in the inclusion trials. Khalili’s meta-analysis included the trials using ALA as the intervention [20]. Another reason is the intervention time. HbA1c reflects the integrated FBG level for the past 3 months, so HbA1c is less affected by short-term n-3 PUFA interventions. The results of this trial showed that FBG and HbA1c decreased significantly after PO intervention compared with FO intervention, which suggested the potential ability of ALA to improve glycemic factors. In accordance with the present results, a 12-week treatment of rapeseed oil (with ALA 9.1%) resulted in a greater decrease in serum HbA1c in the intervention group compared to control group [30]. Another study showed that flaxseed intake could reduce blood glucose and insulin levels and increase insulin sensitivity in overweight and obese people with pre-diabetes [31]. Besides, Khalili et al’s meta-analysis found that there was the significant decrease of glucose after n-3 PUFA intake and the suggested dose and duration for T2DM patients was 1–2 g/d for more than 8 weeks [20]. This meta-analysis included studies administrating not only marine-based n-3 PUFA but also plant-based n-3 PUFA. Perilla oil is a common plant source of n-3 PUFA with rich ALA. ALA promotes glycolipid metabolism [32, 33]. Erythrocytic ALA levels were negatively correlated with T2DM risk in participants with low genetic risk [34]. The large-scale findings also suggested the inverse association of ALA but no convincing association of EPA and DHA with glucose in T2DM [35].

ALA can be converted into EPA, DPA and DHA in specific tissues through the elongation and desaturation enzymatic machinery [36, 37]. However, the conversion efficacy is limited [38, 39]. ALA is the essential precursor of n-3 PUFA that is mainly oxidized to acetyl coenzyme A in the mitochondria, with only a tiny part being transformed into DHA [40]. Through the endoplasmic reticulum and peroxisome processing, ALA does not lead to the accumulation of total omega-3 index [38]. The exact mechanisms underlying the regulation of FBG by ALA remains elusive, but there is evidence suggesting that the receptors involved probably differ from ALA, EPA and DHA. The in vitro study indicated that ALA may induce insulin secretion from the β-cells via direct actions on the pancreas as well as via a GLP-1-mediated indirect mechanism.

The erythrocytic fatty acids compositions were detected. As biomarkers for disease risk and reliable biological indicators of dietary intake, erythrocytic fatty acids are reliable indicators for increased n-3 PUFA intake [41, 42]. After n-3 PUFAs intake, erythrocytic n-3 PUFAs levels were shown to be significantly elevated after the washout period [43]. The omega-3 index is an objective marker for dietary n-3 PUFA intake. Erythrocytic PUFA levels and omega-3 indices reflect the relative n-3 PUFA composition in the main organs [44]. Due to the 90-day turnover rate for erythrocyte, erythrocyte fatty acids composition is likely to be more representative of long-term dietary intake, compared with the fatty acids composition of plasma and serum, which response quickly after n-3 PUFA intake [43]. Previous studies found that fish oil consumption sufficiently increased erythrocytic DHA, DPA, EPA and total n-3 PUFAs levels [45, 46]. Flaxseed oil supplementation (2.4 or 3.6 g/d) has also been shown to significantly increase ALA, EPA and DPA levels [33]. Because of the comparatively low ALA dose (2.0 g/d) in this study, erythrocytic EPA contents in the PO group were lower compared to FO and LFO groups. It has been shown that erythrocytic EPA and omega-3 indices remained unaltered after administering ALA (4 g/d) for 6 weeks [47]. ALA is not a sufficient source for increasing total EPA and DHA, especially among people with high-fat diet habits [48]. However, consumption of marine products exhibited a positive correlation between erythrocytic EPA and DHA levels. Food or supplements that are rich in EPA and DHA can explain half change of n-3 PUFA in the body [49].

Erythrocytic membrane fatty acid patterns could predict the development of T2DM [50, 51]. Levels of specific fatty acids that denaturize enzyme activities in the erythrocytes have been associated with T2DM onset. These fatty acids are considered to be significant biomarkers for poor T2DM outcomes [9, 52, 53]. Long-chain n-3PUFA levels are low in diabetic patients [54]. A significant relationship between EPA or DHA with T2DM risks was not established in eight European countries. However, an inverse association was obtained between ALA and T2DM incidents [35]. Among Asian countries, a Chinese study documented an inverse association between EPA/DPA contents and risks for T2DM. The study did not find any associations between DHA and risks for T2DM [11]. The detailed mechanisms, causal pathways for EPA, DHA and T2DM risks warrant further studies. A possible mechanism is that a high EPA status leads to improving glucagon like peptide 1 release that promotes insulin secretion by the β-cells [55]. In this study, erythrocytic DPA concentrations after PO administration were significantly elevated compared to the other two groups. This result is in tandem with a previous finding [56]. By the fatty acid elongase 2 enzyme, the increased amounts of EPA resulted in DPA changes, which may act as long chain n-3 PUFA reservoirs in humans, incorporated into lipids components [43, 57, 58].

The association between n-6 PUFA and T2DM is heavily influenced by the race, ethnicity, gender or basal levels of n-6 PUFA in the body. There has been a cross-sectional study conducted in China which indicated that higher n-6 PUFAs status was associated with lower risk of T2DM in male [59]. In one Multi-Ethnic Study, biomarker levels of LA were inversely and AA were positively associated with incident T2DM patients. Analyzing results including twenty prospective cohort studies from 10 regions indicated that high LA concentrations related to a 43% lower risk of T2DM, whereas AA levels were not associated with T2DM [60]. In the EPIC-InterAct study, AA also is not associated with T2DM, while intermediate metabolites 18:3 n6, 20:3 n6 are positively associated [35]. Particularly, a combination of fatty acids, especially characterized by high levels of LA, may be potentially more important in T2DM prevention than individual fatty acid or fatty acid subclasses [61]. Besides long-chain PUFA, some SFAs have been shown to exhibit positive associations with T2DM incidents. Higher proportions of C15:0 have been correlated with low risks for T2DM [50]. Other studies have documented that higher C16:0, C17:0 [13], C18:0 and C20:0 are associated with increased risks for T2DM while there were no association between C14:0 and C24:0 and risks for T2DM [62]. Two prospective studies found inverse associations between plasma long chain SFA with T2DM [63, 64]. A previous study did not find any relationship between erythrocytic long chain SFA with T2DM [52]. Therefore, more evidence is needed for evaluating the associations between SFAs status and T2DM. In this trial, FO did not reduce the fast glucose levels while C17:0 and C20:0 were significantly elevated in FO group versus PO and LFO groups.

### Strengths and limitations

This study provided the first direct comparison of EPA, DHA and ALA effects on erythrocytic membrane fatty acid and glucose metabolism in T2DM patients. Besides, the sample size of this trial provided sufficient power. Several sources of potential bias were present. First, rates of *loss* to follow-up were 16.7% for 3 months, and pre-protocol analyses were carried out in all of outcomes, which may cause overestimation. Second, trials implement blinding of patients and outcomes assessor, the assessors who gathered the information and analysts were not fully blinded. The limitations include; first, most participants were overweight and with higher blood pressure. Therefore, the finding of present study may not apply to diabetic patients with normal weight and blood pressures. Second, due to the end-point measurement point, fatty acid composition changes during the intervention could not be determined. Third, common edible oils such as corn oil were not used as the placebo control in the present trials. The beneficial effects of FO on glucose and lipid profiles have been proved in our previous studies. The focus of this study lies in the comparison of the influences of perilla oil with that of fish oil. Fourth, most of participants took oral hypoglycemic agents or injected insulin, which probably was a potential source of bias, although the diabetes treatments had no significant difference among three groups and were not likely to affect the overall results. Finally, because of the small numbers of participants in all groups, a subgroup analysis would have produced unreliable results.

## Conclusion

Perilla oil supplementation decreased glucose levels while fish oil supplementation reduced serum TG levels. Erythrocytic ALA, DPA and total n-6 PUFAs were elevated after perilla oil administration. Erythrocytic EPA, DHA, total n-3 PUFAs and omega-3 index were highly elevated in the FO group, followed by the LFO group. Marine-based and plant-based n-3 PUFAs exhibit different effect on erythrocytic fatty acid composition and regulate glycolipid metabolism. Careful attention should be paid to evaluated T2DM patient’s complications, FBG, TG levels and eating habits when translated into clinical applications of fish oil and perilla oil.

## Data Availability

The datasets used and/or analysed during the current study are available from the corresponding author on reasonable request.

## Abbreviations

ALA: Alpha-linolenic acid
Apo A1: Apolipoprotein A1
Apo B: Apolipoprotein B
AS: Atherosclerosis
BMI: Body mass index
CVD: Cardiovascular diseases
DBP: Diastolic blood pressure
DHA: Docosahexenoic acid
DPA: Docosapentaenoic acid
EPA: Eicosapentaenoic acid
RBCm: Erythrocyte membrane
FO: Fish oil
FFA: Free fatty acids
GC: Gas chromatography
ALT: Glutamic pyruvic transaminase
AST: Glutamic oxalacetic transaminase
HbAlc: Glycated haemoglobin
IL-6: Interleukin 6
LFO: Linseed and fish oil
LPa: Lipoprotein a
MUFA: Monounsaturated fatty acids
n-3 PUFA: Omega-3 polyunsaturated fatty acid
PO: Perilla oil
SFA: Saturated fatty acids
SBP: Systolic blood pressure
TG: Triglyceride
TC: Total cholesterol
T2DM: Type 2 diabetes
TG: Triglycerides
